# Benchmarking survival machine learning models for 10-year cardiovascular disease risk prediction using large-scale electronic health records

**DOI:** 10.1177/20552076251408534

**Published:** 2026-01-22

**Authors:** Tianyi Liu, Andrew Krentz, Lei Lu, Yanzhong Wang, Vasa Curcin

**Affiliations:** 1 School of Life Course & Population Sciences, 4616 King's College London, London, UK; 2 Metadvice, 45 Pall Mall, St James's, London, UK

**Keywords:** Cardiovascular diseases, machine learning, electronic health records, risk prediction, survival analysis

## Abstract

**Objective:**

To evaluate the performance of machine learning (ML)-based survival models for 10-year cardiovascular disease (CVD) risk prediction using large-scale electronic health records (EHRs). The study benchmarks these models against the QRISK3 score and conventional Cox proportional hazards (CoxPH) models currently used in UK primary prevention, with the aim of assessing their potential to capture complex risk patterns beyond traditional approaches.

**Methods:**

This study utilized individual-level data from the CPRD Aurum, covering 40 million UK primary care records from 2011 to 2021. A total of 469,496 patients aged 40–85 was analysed. Predictor variables were selected based on QRISK3 definitions, with additional phenotyping for comorbidities and pre-stratified risk scores. ML models, including deep neural networks (e.g., DeepSurv and DeepHit) and ensemble survival models (e.g., random survival forest [RSF] and gradient boosting), were developed for CVD risk prediction. Model performance was assessed using calibration and discrimination metrics, with ‘spatial external validation’ conducted using a London-held dataset.

**Results:**

A total of 849,651 records were analysed, including 117,421 for ‘spatial validation’ and 732,230 for development. QRISK3 scores effectively differentiated CVD patients, particularly among females, showing stronger predictive performance. Ensemble methods and neural networks outperformed CoxPH models, with RSF achieving the best discrimination and calibration: AUROC values of 0.738 (95% CI: 0.723–0.752) for males and 0.778 (95% CI: 0.762–0.793) for females, with Brier scores of 0.088 and 0.055.

**Conclusion:**

ML models enhance CVD risk prediction, outperforming conventional approaches in calibration and discrimination. Integrating pre-stratified risk scores further improves performance, highlighting the value of augmenting tools like QRISK.

## Introduction

Cardiovascular disease (CVD) remains the leading public health challenge worldwide and in the UK, affecting 7.6 million individuals as of 2024. In 2022, CVD accounted for 26% of all UK deaths, resulting in approximately 174,884 fatalities.^1^

Additionally, major modifiable risk factors for CVD often lack sufficient patient awareness. In the UK, an estimated 6–8 million people have undiagnosed or uncontrolled high blood pressure (BP), and 850,000 people have undiagnosed diabetes. Approximately half of adults have cholesterol levels above the recommended guideline.^1,2^

Regular screenings and timely counselling with medical interventions can address behavioural risk factors, reduce CVD onset and severity, improve health outcomes, and decrease health inequalities.^3^ In the UK, individuals aged 40 and above are routinely invited by their general practitioner (GP) for a CVD risk assessment every five years as part of a general screening strategy, while those aged 25 to 84 with specific risk factors, such as type 2 diabetes, are assessed using the risk score to evaluate their 10-year CVD risk.^4^

Several CVD risk scores have been developed to estimate 10-year CVD risk in primary care for primary prevention. The QRISK3^5^ and Pooled Cohort Equation (PCE)^6^ are widely used in the UK and USA, respectively, guiding statin prescriptions and other therapies in asymptomatic individuals. These models are recommended by guidelines such as National Institute for Health and Care Excellence (NICE)^4^ in the UK and American College of Cardiology/American Heart Association (ACC/AHA)^7^ in the USA.

Researchers have enhanced these algorithms by adding new predictors and conducting external validations.^8,9^ Despite both using Cox proportional-hazard models (CoxPH), some studies suggest these algorithms may misestimate risks for certain subpopulations, leading to controversial outcomes for minor ethnicity groups.^10,11^

Machine learning (ML), including deep learning (DL), is pivotal in the evolving field of CVD primary prevention and management, employing sophisticated algorithms to analyse diverse datasets and uncover complex patterns that traditional methods may overlook. ML leverages both structured and unstructured electronic health records (EHRs) to discern nuanced correlations within clinical data.^12,13^

Recent studies indicate that ML/DL techniques provide superior predictive capabilities for long-term CVD risk, specific cardiovascular events, and time-to-event (TTE) analysis, often surpassing traditional algorithms such as QRISK and Atherosclerotic CVD (ASCVD) risk calculators in both discrimination and calibration.^14^ Integrating conventional risk scores into ML algorithms could further enhance performance and ensure that established, guideline-endorsed methods are effectively leveraged for primary prevention.^15–17^ Moreover, ML models demonstrate an improved ability to accommodate patient heterogeneity, particularly among individuals with comorbid conditions.^12^

Our objective is to develop and benchmark ML models for TTE analysis using longitudinal EHR data, with the ultimate aim of informing the development of risk scores that could be implemented in primary care settings to support real-world clinical decision-making.

Specific goals include:

1. Stratifying individual CVD risk using QRISK3.
2. Developing ML algorithms, including ensemble and neural network survival models, to predict 10-year CVD risk.
3. Comparing and validating model performance in terms of both discrimination and calibration.

To support transparency and reproducibility, we leveraged EHR data with phenotyping techniques to ensure the relevance of predicted outcomes, and systematically documented key technical procedures including variable definitions, data harmonization, and hyperparameter optimization. Model development and reporting were guided by the TRIPOD + AI^18^ framework. By adhering to these principles, we aim to produce clinically grounded, methodologically robust, and replicable ML-based survival models that advance the field of CVD risk prediction.

## Methods

This study is conducted in accordance with the guidelines outlined in the TRIPOD + AI statement,^18^ an extension of the TRIPOD statement specifically for AI/ML prediction models (Supplementary file).

### Study design and participants

This study employs a prospective open cohort design using retrospective individual-level data from UK EHRs, specifically the CPRD Aurum (study registration protocol No: 21_000346). CPRD provides longitudinal, routinely collected de-identified patient data from primary care GPs that use Egton Medical Information Systems (EMIS) Web^®^, an electronic patient record system.

The CPRD Aurum,^19^ updated to the January 2022 version 2.7, is a comprehensive EHR dataset widely used in cardiovascular research and represents a suitable source for developing ML-based risk prediction models for primary prevention. This dataset includes over 40 million research-acceptable patients, with current patients representing approximately one-fifth of the UK's population and covering 1359 GP practices, equating to 16.62% of all UK GPs. The median follow-up time for currently registered patients is 8.74 years, ensuring a comprehensive longitudinal dataset. Further information regarding this dataset can be found in the data specification document available on the CPRD website.^20^

We included a stratified random sample of 469,496 patients aged 40 to 85 years, registered between 2011 and 2021 in England, from the CPRD Aurum database. Sampling was stratified by GP and calendar year to ensure temporal and geographic representativeness. This approach was adopted due to the large size of the full Aurum population which would pose considerable computational challenges for model development, especially when training DL models. The source data consisted of eight structured components: ‘patient’, ‘practice’, ‘staff’, ‘consultation’, ‘observation’, ‘problem’, ‘referral’, and ‘drug issue’ files. These were linked using CPRD-provided unique patient identifiers to reconstruct complete longitudinal primary care records for each individual.

**Figure 1.**
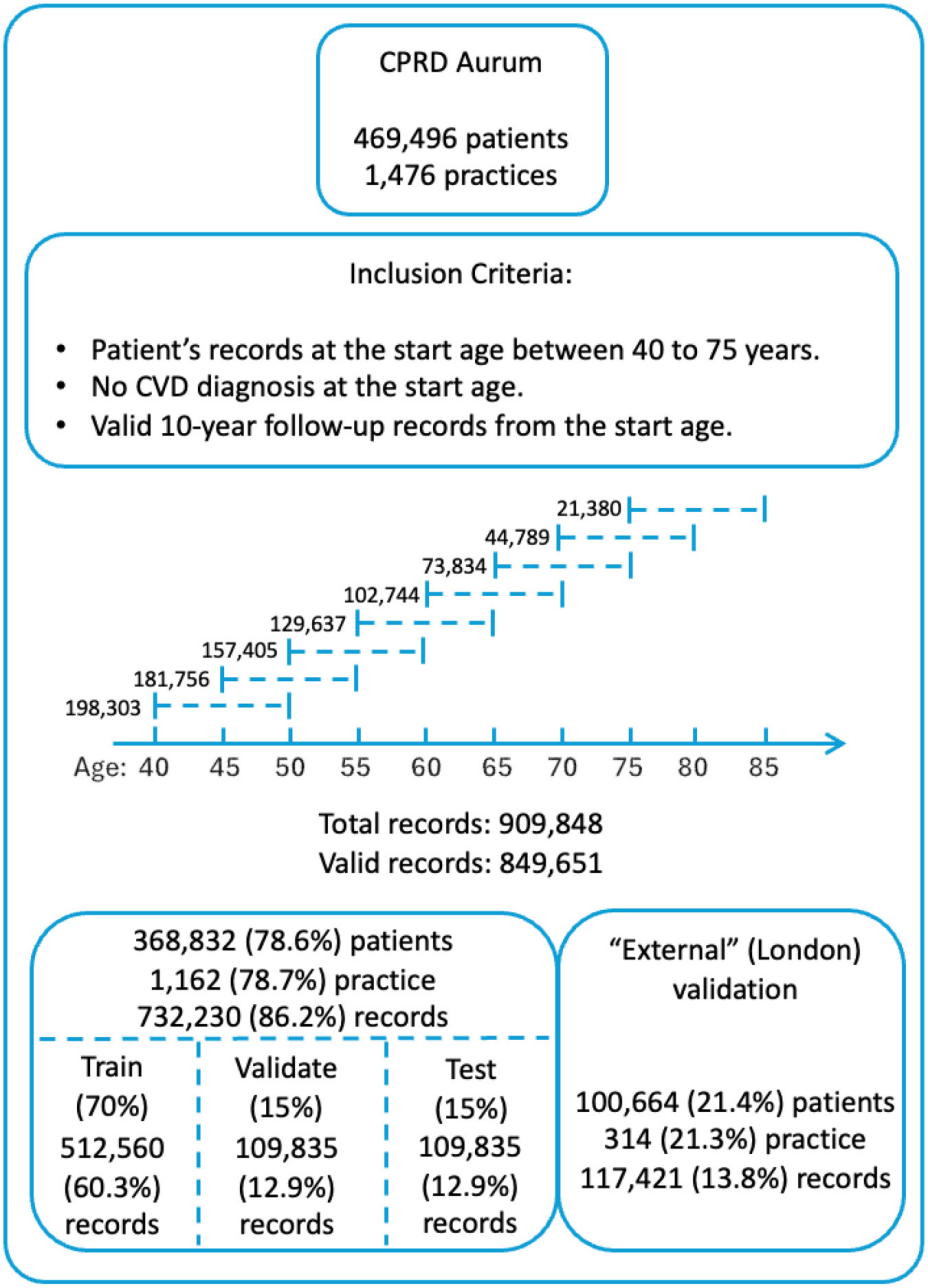
Workflow for study population and records extraction.

From the given dataset (Figure 1), we first conducted a validity check for individual patients to ensure they had an eligible 10-year follow-up horizon with accurate registration, consultation, and clinical observation dates. We then extracted the required features for patients with valid records that included any 10-year duration, starting from ages 40 to 75, in 5-year intervals. This approach allows participants in our study to have multiple entries, provided they are free from CVD at the year of interest, treating these entries as independent 10-year records.

We used a multi-entry cohort design with 5-year intervals to include individuals aged 40 to 85 at multiple time points. This setup allows the model to learn from a broader age range and across different life stages, while increasing the number of usable 10-year follow-up windows. It also ensures appropriate handling of censoring within each prediction horizon, rather than excluding individuals with shorter follow-up. The design reflects how cardiovascular risk is assessed in clinical practice, where patients may undergo repeated risk evaluations over time. In line with this, UK NICE guidelines^4^ recommend reassessing risk every 5 years in adults aged 40 to 85. Conceptually, this approach is consistent with landmark modelling methods,^21^ where individuals contribute multiple fixed-horizon prediction episodes anchored at different time points. Consistent with this framework, we did not model longitudinal trajectories directly but instead constructed multiple baseline entries, mirroring how risk prediction tools are reapplied in practice.

In 2023, approximately 11% of UK adults were living with CVD, with an annual incidence of 1.5 per 1000 for major cardiovascular events such as stroke or myocardial infarction.^22^

We confirmed that the sample size was sufficient for model development by applying the framework proposed by Riley et al.,^23^ which considers shrinkage, calibration, and precision criteria for prediction modelling (Supplementary file). Our final cohort substantially exceeded the minimum required sample size and event count, ensuring stable risk estimation and low risk of overfitting over a 10-year prediction window.

### Predictor variables and cardiovascular disease outcomes

We used the same set of risk predictors as published by QRISK3,^5^ as shown in Table 1. CPRD contains demographic characteristics, diagnoses, symptoms, drug prescriptions, and dosages, using MedCode ID as unique identifiers for labelling medical terms and codes, primarily captured using the EMIS, which is a widely adopted EHR system in UK GP. MedCode ID can be mapped to other controlled clinical terminologies, including Read Code and SNOMED CT.

**Table 1.** Summary of predictor variables and cardiovascular disease outcomes in QRISK3 and expanded stratified risk set.

	QRISK3 set	Expanded stratified risk set
Continuous predictors		
**Body mass index (kg/m²)**	Y	Y
**Systolic blood pressure (mmHg)**	Y	Y
**s.d. of systolic blood pressure**	Y	Y
**Total/High-density lipoprotein ratio**	Y	Y
**Townsend score**	Y	Y
**Risk score (%)**		Y
Categorical predictors		
**Ethnicity (9 categories)**	Y	Y
**Smoking status (5 categories)**	Y	Y
**Risk group (4 categories)**		Y
Binary predictors		
**Type 1 diabetes mellitus**	Y	Y
**Type 2 diabetes mellitus**	Y	Y
**Chronic kidney disease stage 3, 4 or 5**	Y	Y
**Family history of coronary heart disease**	Y	Y
**Atrial fibrillation**	Y	Y
**Erectile dysfunction**	Y	Y
**HIV/AIDS**	Y	Y
**Migraine**	Y	Y
**Rheumatoid arthritis**	Y	Y
**Systemic lupus erythematosus**	Y	Y
**Severe mental illness**	Y	Y
**Antipsychotic**	Y	Y
**Corticosteroid**	Y	Y
**Treated hypertension**	Y	Y
Cardiovascular disease outcomes		
**Coronary heart disease**	Y	Y
**Myocardial infarction**	Y	Y
**Stroke**	Y	Y
**Transient ischemic attack**	Y	Y
**Abdominal aortic aneurysm**	Y	Y
**Peripheral artery disease**	Y	Y
**Heart failure**	Y	Y
**Angina**	Y	Y

NB: “Y” indicates that the predictor or outcome is included in the respective set.

We utilized the phenotype definitions available in the HDRUK Phenotype Library,^24^ which include demographic information, measurement procedures, and drug medications. For the diagnosis of comorbidities, we used the phenotype collection from CALIBER.^25^ Supplementary Table 1 shows the descriptions and corresponding ICD-10 codes used to identify comorbidities. We also documented all the MedCode IDs used to identify predictor variables, mapping them to other controlled terminologies for cross-quality checks, future updates, and transparency (Supplementary file).

We maintained one set of predictor variables based on the exact QRISK definitions. Additionally, we included a separate set incorporating the pre-stratified risk score in both continuous and categorical formats (Table 1) to evaluate whether this approach could improve performance. No further feature selection was performed, as all predictors were pre-specified by the validated QRISK model, ensuring consistency and fairness in model comparisons.

For variables that remain constant during the records (gender, ethnicity, family history, deprivation), we extracted them whenever they existed in the records. For variables that needed to assert the status at the year of entry, such as smoking status, we extracted them within a certain acceptable range (within 6 months before and after the year of entry). If no recent smoking status records were within the acceptable range, we identified the individual as a former smoker if there was a previous history before the year of entry.

For relevant medication use including anti-hypertensive, corticosteroid, and antipsychotic medications, we used the same approach if records showed they existed within the acceptable range around the start year. We labelled them as existing if they appeared at any time in previous records. For disease comorbidities, we labelled them as existing if they occurred in previous records before the start year within a half-year acceptable range. We assumed that no records indicated no disease occurrence rather than missing data.

For continuous variables like body mass index (BMI), systolic blood pressure (SBP), standard deviation (s.d.) of SBP, and total cholesterol/high-density lipoprotein (HDL) value, if no available readings were within the acceptable range, we used previous records to estimate them using linear regression if there were more than three readings within 2 years before and after the acceptable range.

We extracted all the above-mentioned QRISK predictor variables based on the start year when the patient was recruited into the data and used them to create two additional predictor variables based on the calculated QRISK score: one in continuous format and one categorized into 0–5%, 5–10%, 10–20%, and 20%+. The threshold selection was based on the 10% 10-year risk threshold used in NICE guidelines^4^ to recommend statin initiation. For individuals with stage 1 hypertension, the same threshold may also inform decisions around initiating antihypertensive treatment,^26^ depending on overall cardiovascular risk.

For this study, in line with the scope of primary prevention, outcomes were defined as first incident CVD events, which are the basis for preventive treatment decisions in clinical practice and consistent with established tools such as QRISK. We used phenotype definitions from HDRUK Phenotype Library^24^ and the CALIBER^25^ to identify relevant diagnoses from CPRD primary care records using MedCode IDs. These codes were mapped to ICD-10 categories for reporting clarity (Supplementary Table 2). Specifically, we captured coronary heart disease (I20–I25), including angina and myocardial infarction; stroke (I63–I66, I69); transient ischemic attack (TIA) (G45–G46); and other cardiovascular conditions such as heart failure (I50), abdominal aortic aneurysm (AAA) (I71), and peripheral arterial disease (PAD) (I73–I74).

We labelled an occurrence as a CVD outcome when one of the aforementioned types of CVD is observed before the end of the 10-year follow-up, with an acceptable range of six months afterward. If no record was found, we assume the condition did not exist rather than considering it as missing data. We also recorded the TTE information for the CVD outcomes.

### Statistical analysis

As previously mentioned, we utilized QRISK3 to pre-calculate the individual CVD risk at the year of entry. First, we preprocess our data by removing outliers using the interquartile range (IQR) method and iteratively imputing the missing data using the multiple imputation by chained equations (MICE) method, with a random forest regressor as the estimator. We then predict the risk using an R programming package^27^ that exactly replicates the QRISK3 algorithms based on the official open-source implementation released by ClinRisk Ltd under the GNU Lesser General Public License (LGPL).

To interpret the results, we compared the difference in risk scores between records with CVD and those without CVD. We evaluated the calibration by calculating the Brier score and producing the calibration curve. We use the c-statistic to measure discrimination performance, with a 95% confidence interval (CI) calculated using the bootstrap technique. Additionally, we compare the baseline threshold selection of risk scores for different age-risk groups separately for both genders and identify the optimal baseline threshold that yields the best F1 score, ensuring a balance between precision and recall.

We selected random survival forest (RSF), gradient boosting survival analysis (GBSA), extreme gradient boosting for survival analysis (XGBS), and two NN-based survival models (DeepSurv,^28^ a deep NN implementation of the CoxPH; and DeepHit,^29^ a DL model designed for survival analysis with competing risks) as potential ML/DL models for training. DeepSurv, as an extension of the CoxPH model, focuses more on modelling time-dependent survival probabilities, which may allow it to better capture overall trends. In contrast, DeepHit is more oriented toward directly predicting the probability of events, making it potentially more sensitive to subgroup-specific characteristics, such as those associated with gender differences. Although DeepHit is capable of modelling competing risks, we implemented it in a single-endpoint setting consistent with the other models. The choice of these models is based on findings from previous systematic reviews, which indicate that ensemble methods and NN outperform other ML models.^30–34^ CoxPH from two Python packages (Lifelines^35^ and scikit-survival^36^) were used as baseline models, with interaction terms included following the QRISK3 structure for both packages. All models are derived and trained separately by gender.

Initially, we removed outliers using the IQR method. For categorical variables, we created dummy variables using one-hot encoding and scaled continuous variables with MinMaxScaler. We applied the MICE method to impute missing data, particularly to accommodate ML classifiers that do not natively handle incomplete inputs.

For the implementation of ensemble-based survival methods, we used the scikit-survival package,^36^ while for NN-based survival models, we used the PyCox package, which is based on PyTorch.^37^ The dataset was randomly split into training, validation, and testing sets with a 70–15–15 ratio (Figure 1).

During the model tuning process on the training set, we employed Optuna,^38^ a hyperparameter optimization framework based on Bayesian optimization and the Tree-structured Parzen Estimator, to identify the optimal combination of hyperparameters for each model, aiming to balance performance and computational efficiency. The performance of each model is reported separately for the training, validation, and test sets, stratified by gender, using the best hyperparameter combination that achieved the highest F1 score on the validation set. Detailed information on the hyperparameter selection process is provided in Supplementary Table 3.

To evaluate and compare model performance, we selected the best-performing model configuration based on hyperparameter optimization. Calibration plots were generated to compare predicted probabilities with observed event rates in the test set, and Brier scores were calculated to assess overall calibration. AUROC scores and their 95% confidence intervals were computed to quantify each model's discriminatory ability and the associated uncertainty. These metrics were then directly compared across models by gender. Internal validity was assessed using 10-fold cross-validation. Additional performance metrics, including accuracy, specificity, and recall, were reported based on the optimal risk threshold identified by maximizing the F1 score.

We additionally applied SHapley Additive exPlanations (SHAP) to the best-performing models to interpret predictor contributions and assess whether the relative importance and direction of association of variables were consistent with established clinical risk factors.

To assess generalizability in underrepresented populations, we performed ‘spatial external validation’ using a held-out dataset comprising patients from GP in London (Figure 1), a region with the highest proportion of ethnic minority groups in the UK (Table 2). This ‘spatial validation’ set was completely unseen during model training and internal evaluation. All performance metrics described above were also reported for the external dataset.

**Table 2.** Baseline characteristics of the study population by gender.

	Male (n = 399,977, 47.1%)		Female (n = 449,674, 52.9%)	
	Train, Validation, Test (n = 344,895)	Spatial Validation (n = 55,082)	Train, Validation, Test (n = 387,335)	Spatial Validation (n = 62,339)
Continuous predictors, mean (s.d.)				
**Age**	50.66 (9.14)	49.78 (9.02)	51.29 (9.47)	50.75 (9.48)
**Body mass index**	27.44 (4.51)	27.06 (4.47)	27.04 (5.52)	27.19 (5.64)
**Systolic blood pressure**	136.61 (17.10)	134.11 (16.77)	132.53 (18.37)	129.87 (18.17)
**s.d. of systolic blood pressure**	10.46 (6.19)	10.12 (5.96)	10.66 (6.09)	10.39 (5.82)
**Total/High-density lipoprotein ratio**	4.00 (1.24)	3.96 (1.22)	3.96 (1.23)	3.89 (1.11)
**Townsend score**	4.61 (2.73)	7.45 (2.55)	4.57 (2.69)	7.36 (2.55)
**Risk score**	8.86 (7.96)	8.85 (8.30)	6.35 (6.56)	6.95 (7.02)
Categorical predictors, %				
**Ethnicity:**				
** White**	93.49	64.78	94.00	65.55
** Indian**	1.49	8.27	1.46	8.26
** Pakistani**	1.02	2.02	0.91	1.62
** Bangladeshi**	0.25	1.37	0.22	1.21
** Chinese**	0.23	0.96	0.28	1.03
** Other Asian**	0.50	4.39	0.47	4.28
** Black Caribbean**	0.49	5.35	0.55	6.66
** Black African**	0.34	6.80	0.29	6.13
** Other ethnic group**	2.19	6.05	1.83	5.26
**Smoking status:**				
** Non-smoker**	44.91	48.29	57.50	64.42
** Ex-smoker**	52.59	49.27	40.34	33.68
** Light smoker (1–9 cigarettes/day)**	0.58	0.66	0.73	0.71
** Moderate smoker (10–19 cigarettes/day)**	1.17	1.07	0.98	0.86
** Heavy smoker (>19 cigarettes/day)**	0.75	0.70	0.44	0.34
**Risk group:**				
** Low risk**	42.14	44.04	58.33	54.78
** Moderate risk**	26.86	25.57	22.31	23.05
** High risk**	21.56	20.69	14.43	16.07
** Extreme high risk**	9.44	9.69	4.93	6.10
Binary predictors, %				
**Type 1 diabetes mellitus**	0.41	0.37	0.27	0.23
**Type 2 diabetes mellitus**	3.55	5.84	2.55	4.36
**Chronic kidney disease stage 3, 4 or 5**	0.79	0.82	1.21	1.11
**Family history of coronary heart disease**	3.68	2.74	4.72	3.41
**Atrial fibrillation**	0.81	0.57	0.43	0.37
**Erectile dysfunction**	4.10	4.26	NA	NA
**HIV/AIDS**	0.06	0.40	0.02	0.12
**Migraine**	2.54	1.91	6.70	5.43
**Rheumatoid arthritis**	0.37	0.32	0.86	0.90
**Systemic lupus erythematosus**	0.05	0.05	0.20	0.28
**Severe mental illness**	0.83	1.49	0.86	1.44
**Antipsychotic**	0.37	0.65	0.37	0.63
**Corticosteroid**	3.25	2.03	4.72	3.21
**Treated hypertension**	7.47	8.03	8.49	9.47
Outcome variables, %				
**Cardiovascular disease**	9.97	8.47	5.97	5.16
** Coronary heart disease**	4.22	3.54	2.14	1.72
** Myocardial infarction**	2.34	1.79	0.89	0.65
** Stroke**	2.11	1.62	1.57	1.35
** Transient ischemic attack**	1.33	0.90	1.06	0.77
** Abdominal aortic aneurysm**	0.42	0.41	0.08	0.09
** Peripheral artery disease**	1.12	0.98	0.58	0.39
** Heart failure**	1.28	1.22	0.87	0.83
** Angina**	3.80	3.31	1.98	1.63

## Results

### Baseline characteristics of study population

We extracted 909,848 records from 469,496 patients across 1476 practices in the England (Figure 1). After a quality check, we retained 849,651 valid records for our model development. We held out 117,421 (13.8%) valid records from practices in London as our ‘spatial validation’ set. For the remaining 732,230 (86.2%) records, we used them as the training, validation and test set by randomly splitting them 70:15:15.

Table 2 presents the characteristics of the baseline training, validation, test set, and the ‘spatial validation’ set, disaggregated by gender. The analysis reveals that most comorbidities and CVD outcomes are more prevalent among males, highlighting the importance of developing gender-specific models. Furthermore, the data indicates a higher proportion of minority ethnic groups in the London held-out set compared to the training/validation/test set. The mean individual deprivation score in the London set is also higher than that of the training and test sets, with men showing slightly higher scores than women. This suggests that selected individuals in the Greater London area experience greater material deprivation compared to those in other parts of the England. The demographic distribution supports the use of the London region as a spatial held-out set for evaluating the model's performance in managing minority ethnic groups.

### Distribution and performance of QRISK3

**Figure 2.**
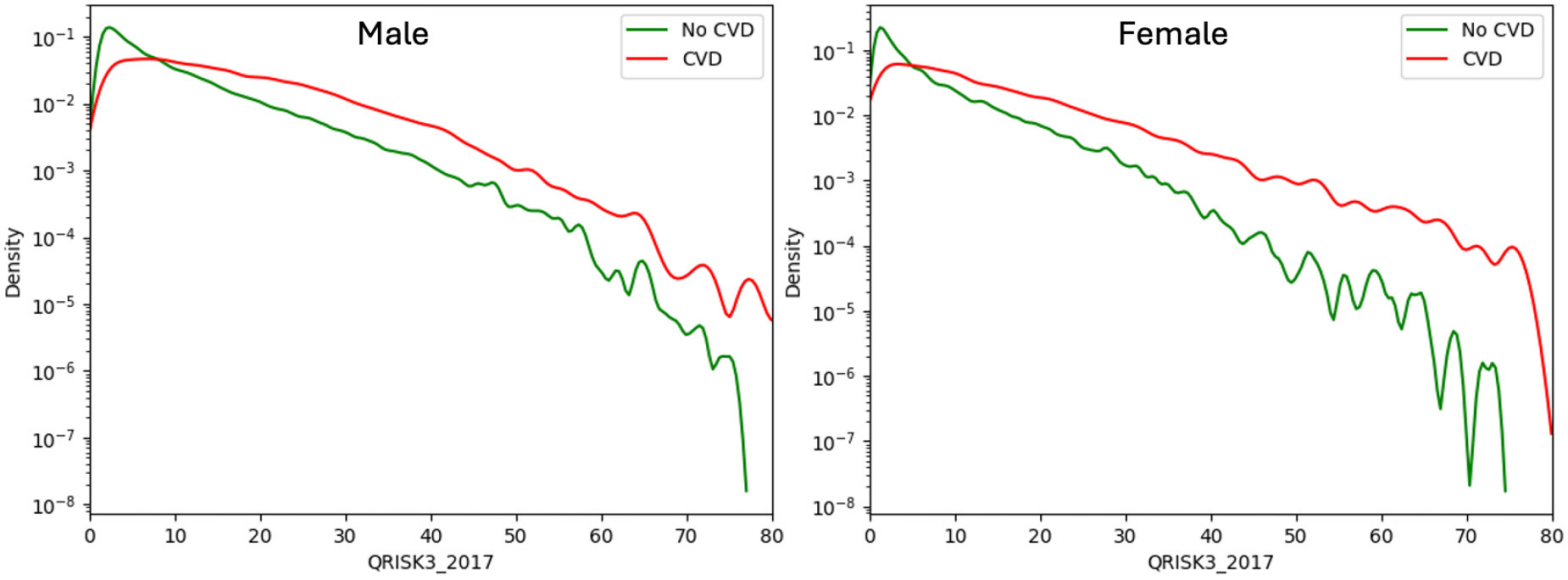
Density plot of QRISK3 scores for patients with and without cardiovascular disease (CVD) by gender.

Figure 2 presents gender-specific density plots comparing QRISK3 score distributions between patients with and without subsequent CVD. Overall, QRISK3 scores effectively distinguish between the two groups, with higher scores linked to greater CVD risk. This separation is more pronounced in females, particularly at scores above 20%, suggesting stronger predictive utility. However, for both genders, scores near 5% show limited discriminative ability.

**Figure 3.**
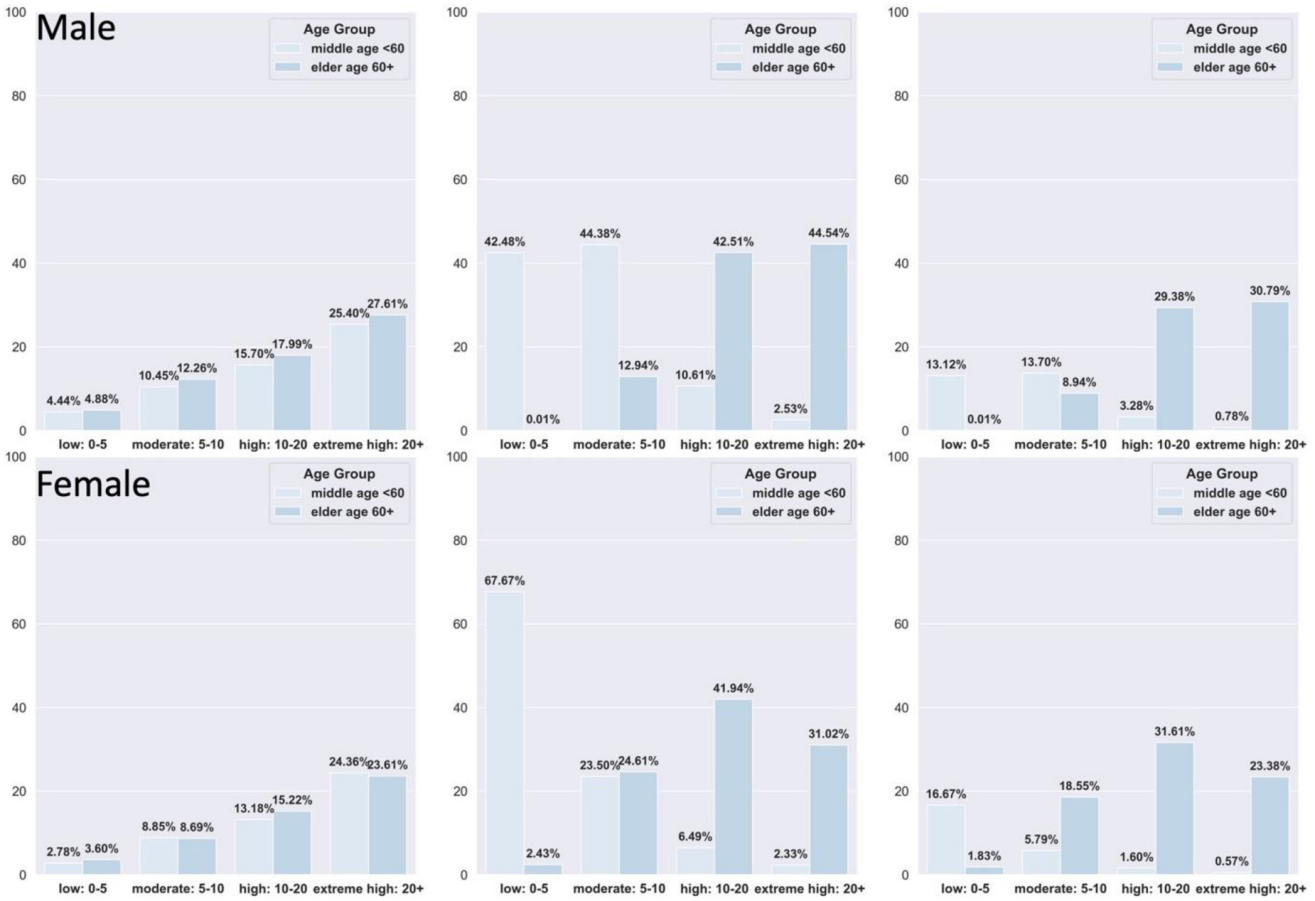
Percentage distribution of patients with cardiovascular disease by age-risk group and gender. Left: Percentage of patients with CVD within each age risk group. Middle: Percentage of CVD patients within each age group, summing to 100%. Right: Percentage distribution of CVD patients across all age risk groups, summing to 100%.

Figure 3 further supports these findings. Many future CVD cases, particularly among females, fall within the low-to-moderate risk groups. Females, especially those under 60, generally have lower risk scores, limiting the score's discriminative power in younger age groups. In contrast, risk scores are more informative in older populations. Males tend to have higher scores overall, and an appropriate baseline threshold to capture most CVD cases may be >10% for males and <10% for females.

**Figure 4.**
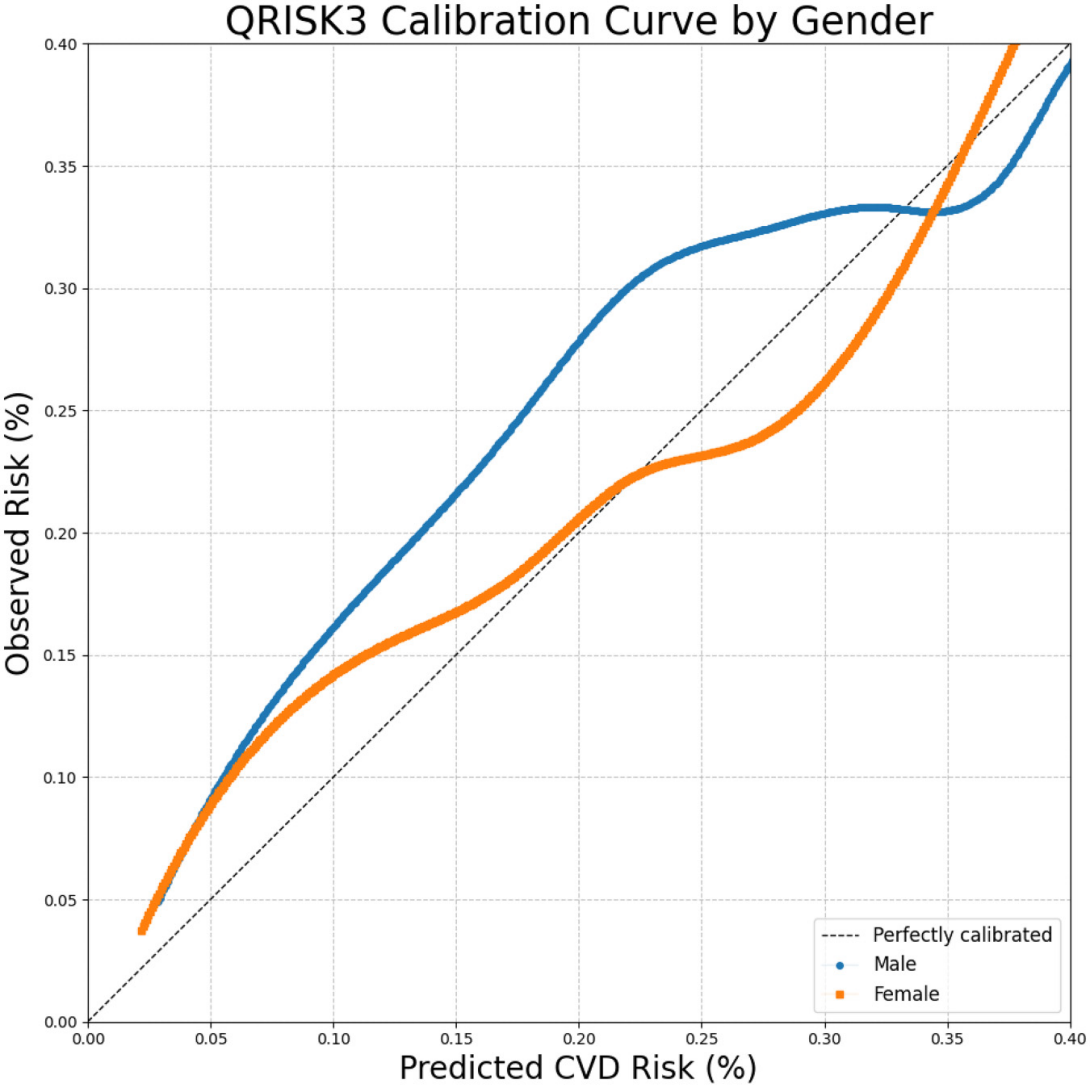
Calibration plot of QRISK3 predicted vs. observed cardiovascular disease (CVD) risk by gender.

As shown in Table 3, the optimal thresholds for QRISK scores were 12.5% for males and 9.3% for females, highlighting the need for lower thresholds in women. Notably, the optimal threshold for the full population matched the 10% NICE guideline, suggesting it may be suitable at the population level but suboptimal for specific subgroups. QRISK performance was slightly better in females (AUROC: 0.747, 95% CI: 0.745–0.749) than in males (AUROC: 0.727, 95% CI: 0.725–0.729), with a lower Brier score in females (0.069 vs. 0.104), indicating better calibration. These findings are also reflected in the calibration plots (Figure 4).

**Table 3.** QRISK3 performance metrics by gender.

	Baseline threshold (%)	Accuracy	Specificity	Recall	F1	AUROC (95% CI)	Brier score
Male	12.5	0.746	0.754	0.532	0.359	0.727 (0.725, 0.729)	0.104
Female	9.3	0.777	0.798	0.529	0.279	0.747 (0.745. 0.749)	0.069
All	10.0	0.770	0.801	0.496	0.304	0.735 (0.733, 0.736)	0.086

### Model predictive performance and comparison

In general, ensemble-based survival methods consistently outperformed CoxPH and QRISK3 baselines across discrimination and calibration metrics, with RSF showing the strongest overall performance. DL models also achieved competitive results, particularly in the female subgroup.

Table 4 displays the performance metrics of five ML-based survival models and two CoxPH baselines using both the original QRISK3 variable set and the expanded stratified risk variable set on the test and ‘spatial validation’ sets. Full results including training and internal validation sets are reported in Supplementary Table 4.

**Table 4.** Performance metrics of machine learning models by gender and variables set.

		Male							Female						
		Baseline threshold (%)	Accuracy	Specificity	Recall	F1	AUROC (95% CI)	Brier score	Baseline threshold (%)	Accuracy	Specificity	Recall	F1	AUROC (95% CI)	Brier score
CoxPH (lifelines)	QRISK3 set	9.35	0.670	0.672	0.657	0.302	0.721 (0.715, 0.727)	0.091	5.74	0.689	0.688	0.708	0.231	0.758 (0.750, 0.765)	0.058
		8.44	0.656	0.654	0.677	0.262	0.723 (0.716, 0.729)	0.078	4.85	0.679	0.677	0.725	0.183	0.766 (0.760, 0.774)	0.045
	Expanded stratified risk set	10.15	0.661	0.660	0.674	0.302	0.724 (0.717, 0.730)	0.091	5.95	0.675	0.671	0.731	0.229	0.760 (0.752, 0.767)	0.058
		9.77	0.658	0.656	0.677	0.264	0.725 (0.719, 0.731)	0.078	5.68	0.692	0.691	0.717	0.188	0.769 (0.762, 0.776)	0.045
CoxPH (sksurv)	QRISK3 set	9.02	0.657	0.655	0.674	0.300	0.721 (0.715, 0.727)	0.091	5.76	0.576	0.699	0.694	0.233	0.757 (0.750, 0.765)	0.058
		8.34	0.659	0.657	0.675	0.263	0.722 (0.716, 0.729)	0.078	4.60	0.677	0.675	0.725	0.182	0.766 (0.759, 0.774)	0.045
	Expanded stratified risk set	10.71	0.676	0.679	0.654	0.305	0.723 (0.717, 0.730)	0.091	6.18	0.682	0.680	0.717	0.229	0.759 (0.751, 0.766)	0.058
		9.77	0.662	0.661	0.671	0.264	0.724 (0.717, 0.730)	0.078	5.36	0.688	0.686	0.717	0.186	0.768 (0.761, 0.776)	0.045
RSF	QRISK3 set	9.32	0.634	0.621	0.735	0.308	0.732 (0.717, 0.746)	0.090	7.36	0.743	0.683	0.683	0.260	0.771 (0.755, 0.786)	0.056
		11.79	0.639	0.637	0.667	0.250	0.704 (0.698, 0.711)	0.080	6.81	0.660	0.659	0.683	0.167	0.730 (0.723, 0.738)	0.046
	Expanded stratified risk set	10.53	0.663	0.659	0.699	0.316	0.738 (0.723, 0.752)	0.088	6.85	0.719	0.720	0.713	0.252	0.778 (0.762, 0.793)	0.055
		12.28	0.638	0.645	0.684	0.254	0.707 (0.701, 0.713)	0.080	7.72	0.700	0.675	0.669	0.181	0.744 (0.737, 0.752)	0.046
GBSA	QRISK3 set	9.59	0.637	0.629	0.696	0.299	0.719 (0.705, 0.734)	0.092	5.30	0.674	0.671	0.710	0.224	0.749 (0.733, 0.766)	0.058
		10.81	0.651	0.651	0.649	0.251	0.705 (0.698, 0.711)	0.082	5.34	0.668	0.666	0.704	0.174	0.740 (0.733, 0.747)	0.047
	Expanded stratified risk set	10.06	0.654	0.650	0.689	0.307	0.728 (0.713, 0.742)	0.091	5.36	0.668	0.664	0.717	0.223	0.751 (0.735, 0.766)	0.058
		11.00	0.659	0.638	0.677	0.264	0.725 (0.719, 0.731)	0.078	5.40	0.682	0.673	0.722	0.184	0.761 (0.753, 0.768)	0.046
XGBS	QRISK3 set	5.10	0.652	0.647	0.697	0.304	0.732 (0.726, 0.739)	0.092	2.00	0.708	0.710	0.683	0.236	0.765 (0.758, 0.772)	0.059
		4.71	0.654	0.654	0.654	0.255	0.708 (0.702, 0.715)	0.080	1.14	0.664	0.662	0.705	0.172	0.739 (0.732, 0.747)	0.047
	Expanded stratified risk set	3.27	0.659	0.655	0.692	0.306	0.736 (0.730, 0.743)	0.095	2.58	0.677	0.674	0.722	0.228	0.762 (0.754, 0.769)	0.059
		3.78	0.652	0.650	0.670	0.258	0.715 (0.709, 0.721)	0.081	2.71	0.670	0.667	0.720	0.178	0.754 (0.746, 0.761)	0.046
DeepSurv	QRISK3 set	10.69	0.658	0.655	0.683	0.303	0.727 (0.721, 0.734)	0.090	6.13	0.681	0.680	0.698	0.224	0.751 (0.743, 0.759)	0.058
		9.47	0.641	0.636	0.687	0.257	0.716 (0.709, 0.722)	0.078	5.26	0.688	0.687	0.711	0.185	0.764 (0.756, 0.771)	0.045
	Expanded stratified risk set	10.15	0.649	0.643	0.695	0.301	0.727 (0.721, 0.733)	0.090	6.98	0.688	0.686	0.706	0.230	0.758 (0.750, 0.765)	0.058
		9.92	0.652	0.649	0.679	0.260	0.722 (0.715, 0.728)	0.079	6.31	0.688	0.687	0.718	0.186	0.765 (0.758, 0.772)	0.045
DeepHit	QRISK3 set	9.96	0.646	0.651	0.704	0.302	0.729 (0.722, 0.735)	0.097	4.69	0.707	0.687	0.691	0.238	0.762 (0.755, 0.769)	0.058
		7.80	0.660	0.659	0.669	0.263	0.722 (0.715, 0.728)	0.079	4.07	0.694	0.693	0.706	0.186	0.766 (0.759, 0.773)	0.045
	Expanded stratified risk set	8.56	0.658	0.628	0.679	0.301	0.725 (0.719, 0.732)	0.098	5.14	0.685	0.674	0.718	0.231	0.763 (0.756, 0.771)	0.058
		7.34	0.653	0.650	0.683	0.263	0.726 (0.719, 0.732)	0.079	5.10	0.682	0.679	0.728	0.185	0.768 (0.761, 0.775)	0.045

NB: In each cell, test set: first value; ‘spatial validation’ set: second value

Overall, during the model development stage, the same ML models using the expanded stratified risk variable set generally outperform those using the original QRISK3 variable set in terms of classification, discrimination, and calibration (Supplementary Table 4).

**Figure 5.**
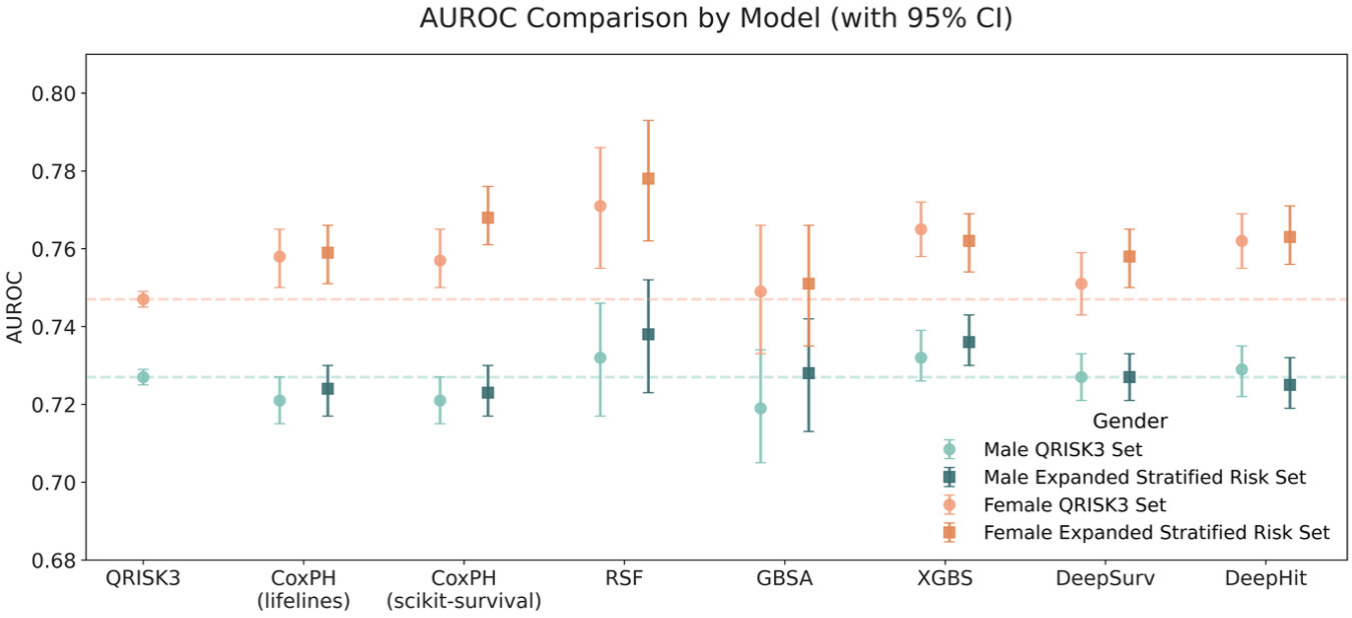
AUROC comparison across models in test dataset by gender (with 95% CI).

Figure 5 shows the AUROC comparison across seven models using both the QRISK3 variable set and the expanded stratified risk set in the test dataset. AUROC scores using QRISK3 are used as benchmark baselines for comparison. Again, these results confirm that models trained on the expanded stratified risk set achieve better discrimination performance than those trained on the QRISK3 variable set.

All five ensemble-based survival methods and the NN-based models demonstrate superior performance in classification and discrimination compared to the two CoxPH baselines in the test set (Table 4, Figure 5), which show nearly identical performance across genders. Specifically, CoxPH (lifelines) achieves an AUROC of 0.724 (95% CI: 0.717–0.730) for males and 0.760 (95% CI: 0.752–0.767) for females, while CoxPH (sksurv) attains an AUROC of 0.723 (95% CI: 0.717–0.730) for males and 0.759 (95% CI: 0.751–0.766) for females. Minor differences are expected due to variations in package defaults such as tie handling and baseline estimation.

Among all models, RSF demonstrates the strongest performance in terms of both discrimination and calibration in the test set, as shown in Table 4 and Figure 5. It achieves an AUROC of 0.738 (95% CI: 0.723–0.752) for males and 0.778 (95% CI: 0.762–0.793) for females. It also has the lowest Brier scores among all models: 0.080 for males and 0.055 for females.

Two other ensemble-based methods, GBSA and XGBS, also perform well in the test set (Table 4). GBSA achieves an AUROC of 0.728 (95% CI: 0.713–0.742) for males and 0.751 (95% CI: 0.735–0.766) for females, with corresponding Brier scores of 0.091 and 0.058, respectively. XGBS reports an AUROC of 0.736 (95% CI: 0.730–0.743) for males and 0.762 (95% CI: 0.754–0.769) for females, with Brier scores of 0.095 and 0.059, respectively.

The two NN-based models perform marginally worse than the ensemble methods in the test set (Table 4). DeepSurv achieves an AUROC of 0.727 (95% CI: 0.721–0.733) for males, slightly outperforming DeepHit's AUROC of 0.725 (95% CI: 0.719–0.732) in the same setting. However, DeepHit performs better in females, with an AUROC of 0.763 (95% CI: 0.756–0.771) compared to 0.758 (95% CI: 0.750–0.765) for DeepSurv.

RSF achieves the best performance among ensemble methods in the training dataset but shows a noticeable decline in the test dataset. Despite this drop, RSF maintains overall strong performance, with consistent results in the validation and test sets, suggesting no signs of overfitting. In contrast, GBSA and XGBS demonstrate more stable performance across the training, validation, and test datasets, with smaller declines from training to testing (Table 4, Supplementary Table 4). The NN-based models, DeepSurv and DeepHit, show slightly lower overall performance compared to ensemble methods but remain competitive. The CoxPH models serve as baselines, showing consistent but lower performance than the ML-based models. These results highlight the superior discrimination ability of ensemble methods such as RSF, GBSA, and XGBS, while also demonstrating their robustness across datasets. Overall, the models exhibit stable performance across the training, validation, and test datasets, with no clear evidence of overfitting for any of the methods evaluated (Table 4, Supplementary Table 4).

**Figure 6.**
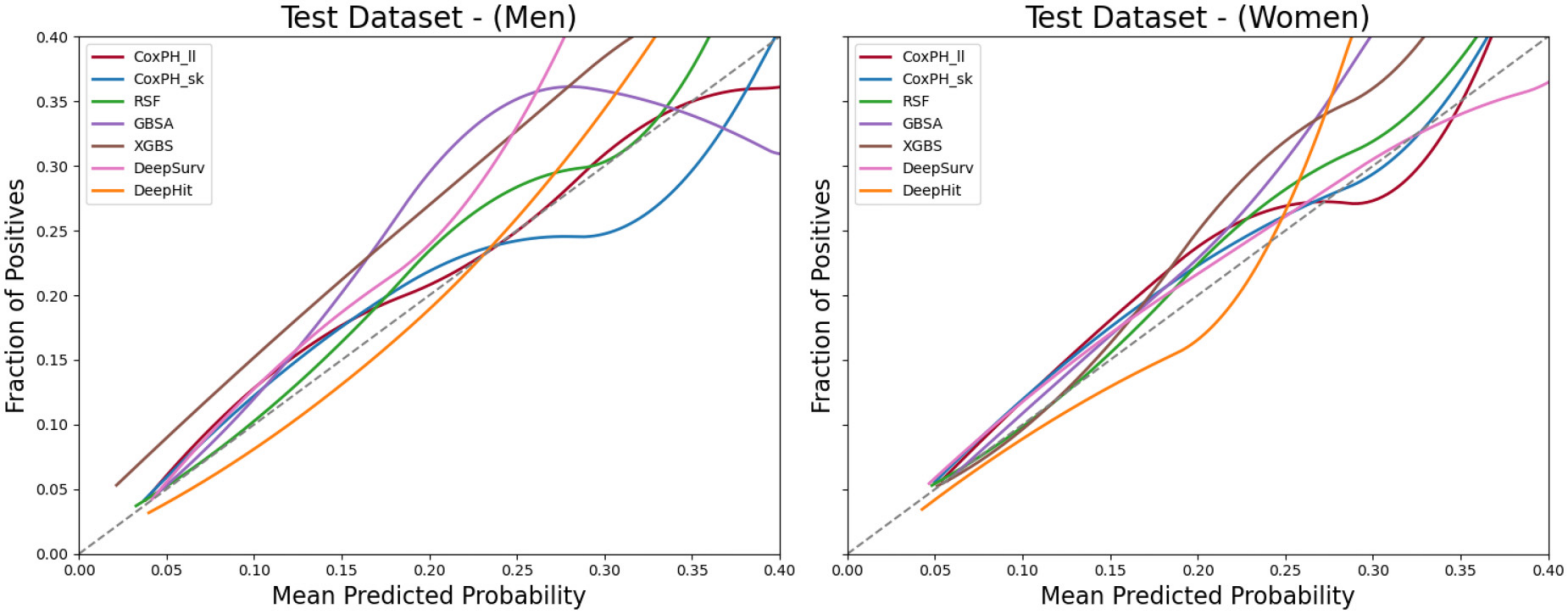
Calibration curves for model performance in test dataset by gender. NB: Calibration plots are truncated at 0.40 to improve visual clarity. Predictions above this threshold are rare and have limited impact on interpretation.

Figure 6 presents the calibration curves for all seven models using the expanded stratified risk variable set in the test datasets. Most models, particularly RSF, align closely with the best-fit line, indicating good calibration. In contrast, XGBS consistently lies above the diagonal, suggesting a tendency to overestimate risk probabilities. This may be attributed to its aggressive gradient boosting optimization, which, while effective for discrimination, can introduce calibration challenges. Other models, including DeepSurv, DeepHit, and the CoxPH baselines, exhibit relatively stable calibration across the test datasets, generally following the best-fit line without substantial deviation. These results indicate that most models are well-calibrated, though the overestimation pattern observed in XGBS warrants further investigation.

In addition, the result tables and plots show that all five ML-based survival models tend to perform better in females than in males in the test dataset. The optimal F1 score–based thresholds for females are also generally lower than those for males. These findings highlight the importance of implementing gender-specific thresholds in ML-based models to improve classification performance in clinical settings, rather than relying on a uniform threshold across genders.

**Figure 7.**
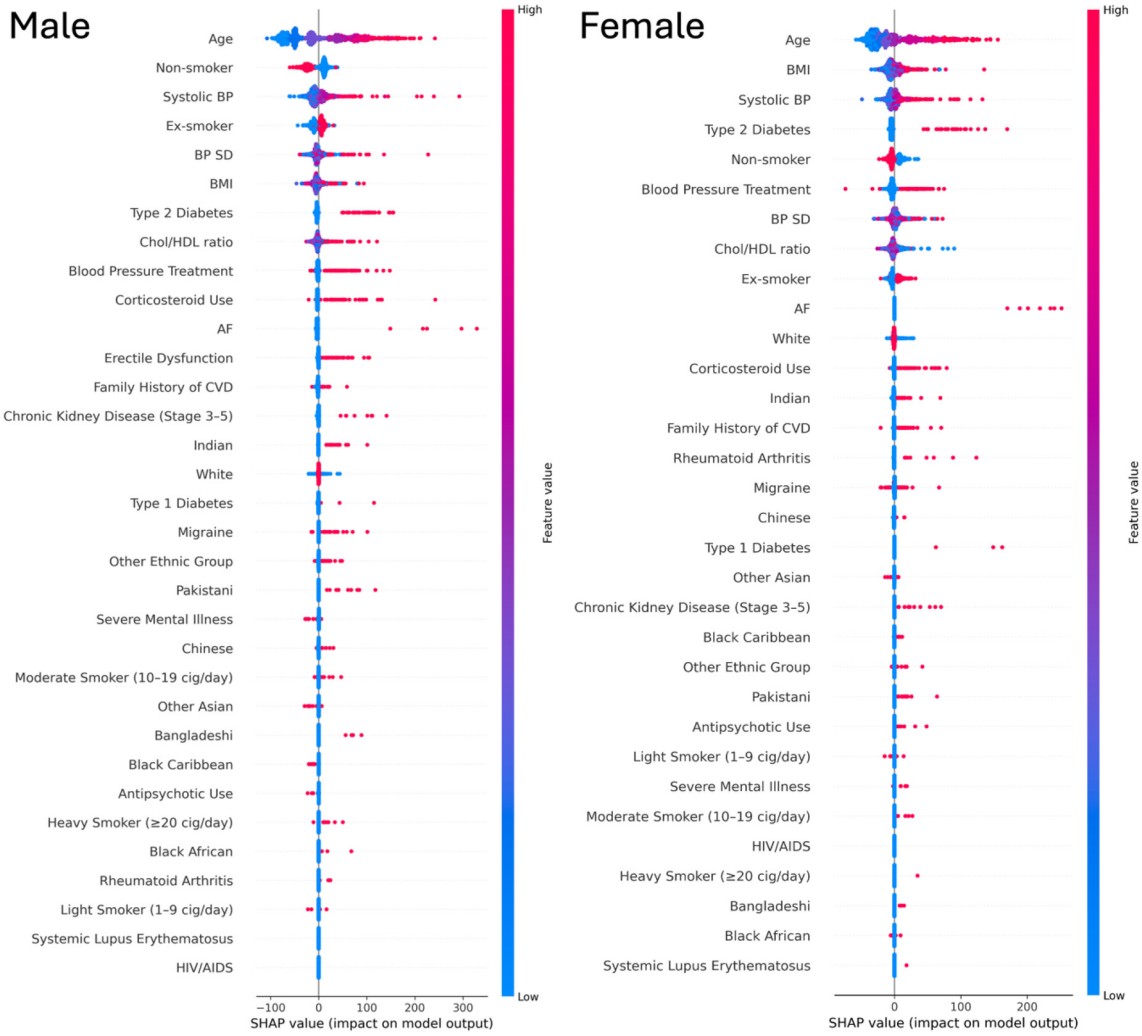
SHAP summary plots of Random survival forest model by gender. NB: Red indicates higher feature values and blue indicates lower feature values. The x-axis shows SHAP values (impact on model output).

To further interpret model predictions, we examined feature contributions using SHAP applied to the best-performing model (RSF). The summary plots (Figure 7) show that age, SBP, and BMI were consistently the strongest continuous predictors of CVD risk in both men and women, with higher values associated with greater risk. Smoking was particularly influential in men, with ex-smokers and increasing categories of current smoking showing progressively higher risk contributions compared with non-smokers. Ethnicity effects were clear, for instance South Asian groups (Indian and Pakistani) showed higher risk contributions. Among comorbidities, diabetes, especially type 2, was the strongest contributor, while AF and CKD also showed significant importance compared with other conditions. BP treatment and corticosteroid use also had notable impact in both genders.

### Model performance in the ‘spatial validation’ set

All models using the expanded stratified risk set demonstrate improved performance compared to those using the original QRISK3 feature set in the ‘spatial validation’ set, as shown in Table 4.

**Figure 8.**
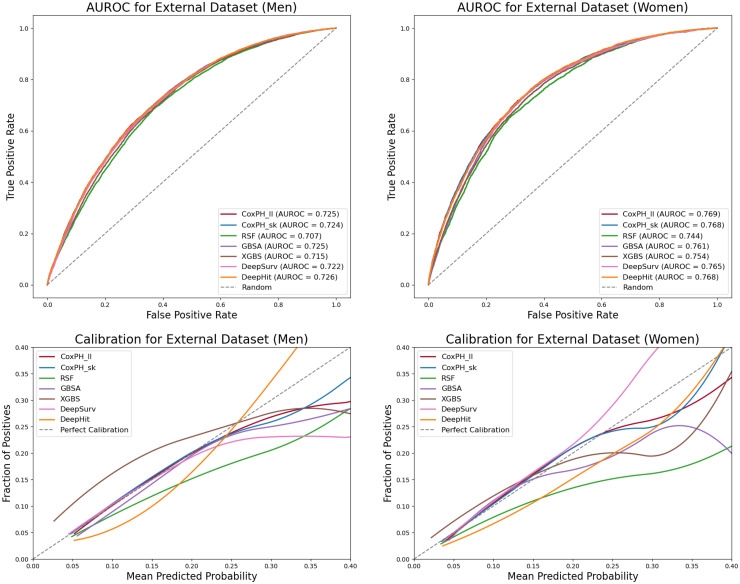
Model performance in ‘spatial validation’ dataset by gender. NB: Calibration plots are truncated at 0.40 to improve visual clarity. Predictions above this threshold are rare and have limited impact on interpretation.

The CoxPH baselines perform consistently well, with stable AUROC values that occasionally exceed those of more complex models. CoxPH (Lifelines) achieves an AUROC of 0.725 (95% CI: 0.719–0.731) for men and 0.769 (95% CI: 0.762–0.776) for women, while CoxPH (scikit-survival) reports 0.724 (95% CI: 0.717–0.730) for men and 0.768 (95% CI: 0.761–0.776) for women. Both methods also show strong calibration (Figure 8), with Brier scores of 0.078 for men and 0.045 for women.

Among the more advanced models, NN–based survival methods (DeepSurv and DeepHit) demonstrate stable performance across datasets, with minimal degradation in the ‘spatial validation’ set. In several cases, these models outperform traditional ensemble methods, highlighting their generalizability.

Within the ensemble models, GBSA performs particularly well in the ‘spatial validation’ set. It achieves the highest AUROC among ensemble methods for both genders, with 0.725 (95% CI: 0.719–0.731) for men and 0.761 (95% CI: 0.753–0.768) for women and maintains reliable calibration (Figure 8).

In general, model performance is slightly better for women than for men in terms of discrimination (Table 4). Calibration curves (Figure 8) for women also show closer alignment with the ideal calibration line, particularly at lower predicted CVD risk levels. These findings underscore the value of gender-specific calibration and thresholding when translating model predictions into clinical decision-making.

Finally, calibration curves (Figure 8) reveal that most models are well-calibrated for lower predicted risks (below 20%) but tend to underestimate actual CVD risk in the higher range (20–40%). This underestimation is especially noticeable in RSF and DeepHit, whose calibration curves remain consistently below the diagonal in the ‘spatial validation’ set.

## Discussion

Using a prospective open cohort design based on retrospective large-scale EHR data, we find that ensemble-based survival methods, particularly RSF, perform well in terms of calibration and discrimination. Numerous model development papers have shown similar results, indicating that these ML models yield better performance.^33,34^ Previous systematic reviews also indicate that ensemble methods may produce superior results.^30–32^

The performance of some of the selected models, particularly RSF, declines in the London held-out dataset, especially for higher predicted risks. This decline is attributed to the different distribution of minority ethnic groups in the validation set. Future efforts should focus on recalibrating for minority ethnic groups. Despite this, the models still show good calibration when the predicted risk is at a low to moderate level below 20%. The hypothesis for this performance drop suggests that predictions still heavily rely on age, with older individuals tending to have higher scores that overestimate the actual CVD risk. In clinical practice, particularly in primary prevention tasks, a higher risk score may not be necessary for decision-making, although achieving accurate predictions across all risk levels is desirable. For primary prevention, it is crucial to identify potential CVD outcomes for patients at an early stage, ensuring the validity of predictions at lower to moderate risk levels.

The increasing use of large-scale comprehensive EHR datasets in model development and validation has become a recent trend.^13^ We observed that recent studies using EHR as data sources for long-term CVD prediction are typically rich in the number of predictors and data size compared to study based on conventional cohort.^39^ Using CPRD as an extensive and diverse EHR data source with longitudinal medical history and diagnoses ensures consistency during model development and maintains data quality and fairness.^40^ Our study underscores the potential of ML on EHR to refine and improve CVD risk prediction.

We utilized QRISK to pre-calculate risk scores before training the ML-based models, aiming to gain deeper insights into individual risk stratification and to leverage the advantages of conventional statistical models. Although QRISK4 has been published recently,^41^ its code is currently unavailable, and it has not yet been adopted by UK guidelines. Therefore, QRISK3 remains the basis of our work.

The AUC/C-statistic performance of QRISK in our study may appear lower than reported elsewhere, likely due to the stricter alignment between our predictors and outcomes over a true 10-year period. However, QRISK demonstrates excellent calibration, which is particularly critical for tasks like this. Our ultimate goal is not merely to surpass the performance of conventional models but to achieve more accurate predictions of patients’ CVD outcomes by integrating their strengths. We also identified studies that incorporated the ASCVD risk score as a predictor in their ML models, achieving improved performance. This highlights the potential of combining conventional and ML-based approaches for better risk prediction.^15–17^

Our analysis showed consistent differences in optimal classification thresholds between males and females, with thresholds typically lower for females, reflecting differences in baseline event rates and predicted risk distributions. It may also be useful to differentiate thresholds for different age groups or set a more personalized baseline, considering additional features. However, this must balance computational cost and generalizability. These thresholds were not intended as clinical cut-offs, but to optimise model performance. While they operate on the same risk scale as clinical guidelines (e.g., NICE's 10% threshold), their purpose is to maximize classification metrics, whereas clinical thresholds consider factors such as cost-effectiveness, feasibility, and policy simplicity. We suggest that male and older age groups may benefit from higher thresholds to indicate moderate risk, but further clinical validation is needed. These findings should be viewed as a step toward clinical translation, which remains outside the scope of this study. Consistent with these threshold differences, the direction of predictor outcome associations in our models aligned with established clinical knowledge. SHAP analysis further showed that the relative impact of several risk factors differs by gender, reinforcing the rationale for gender-specific modelling and thresholding. Our model identified age, SBP, and BMI as strong predictors of CVD risk. Notably, the South Asian groups (Indian and Pakistani) showed higher risk contributions of CVDs. These findings are consistent with previous literature.^42,43^ However, the sex-specific variations of these associations warrant further investigation to better understand potential gender and ethnic disparities in CVD risks.

Developing and comparing ML models is common, but these efforts are often limited to specific methodologies, lacking external validation or practical implementation.^44^ Unlike other model comparison papers or systematic reviews, which often use different validation approaches between ML and classical statistics, making results hard to interpret, our work focuses on fairly comparing widely used ML-based survival models that handle censoring and TTE outcomes. We ensure that our models are both clinically relevant and technically robust. While the “black box” issue of ML models persists, we prioritize transparency in mapping predictors and CVD outcomes, as well as in replicating and validating our models. Adhering to the TRIPOD + AI guidelines,^18^ we used standardized methods and terminology to define and extract predictor variables and outcomes, employing validated, publicly available phenotyping to enhance clinical relevance. By reporting detailed mapping, techniques, and hyperparameters, we ensure transparency and replicability, reinforcing model robustness.

### Limitations

The primary limitation of our study is the absence of a clinical utility analysis. For our work to be practically applied and potentially replace conventional risk scores, it must demonstrate clinical applicability.^45^ Moreover, evaluating ML-based CVD risk scores is crucial to ascertain their substantial benefits for clinical decision-making and consequent improvement in patient outcomes. This evaluation should encompass considerations of ethical implications, legal aspects, economic cost-effectiveness, and the impact on health inequality.^46^

Our study design may introduce potential sampling and measurement bias, as multiple records from the same individuals were included. This was an intentional choice to simulate real-world clinical scenarios, where patients’ cardiovascular risk is typically reassessed every five years. The resulting structure is conceptually aligned with landmark modelling approaches,^21^ which use repeated entry points and fixed prediction windows to capture evolving risk over time. Consistent with this framework, we did not model full longitudinal trajectories directly but instead focused on repeated baseline entries, mirroring how risk tools are reapplied in clinical practice. While more advanced time-series approaches could provide additional insights, these fall outside the scope of the present benchmarking study. To mitigate bias, we applied a consistent 5-year re-entry framework and constructed fixed 10-year prediction windows for each entry. We also incorporated an acceptance range for predictor and outcome definitions (e.g., a six-month margin) to accommodate potential underreporting or right censoring in EHRs. Given the long prediction horizon, small temporal gaps are unlikely to meaningfully affect the modelling of an individual's CVD risk trajectory.

We focused solely on CVD outcomes recorded in GP within primary care, consistent with our primary prevention goals where the aim is to estimate the risk of first incident events in order to guide early detection, management, and intervention. Mortality data can provide valuable supplementary information, but its inclusion is not essential for this benchmarking objective, which was designed to evaluate baseline prediction of incident CVD events.

Furthermore, our dataset is specifically constructed with a 10-year gap between predictors and outcomes to ensure robust temporal alignment. While this approach may result in lower classification performance compared to other studies, we observed strong calibration, which is more critical for ranking risk and ensuring actionable predictions.

We did not include individuals under the age of 25, despite the trend of earlier CVD onset, as we believe younger age group warrant separate analysis. Our study focuses on middle-aged individuals, where risk trajectories are more distinct and clinically relevant.

Despite their predictive advantages, ML-based models face practical limitations that hinder clinical integration. They are more susceptible to performance heterogeneity due to variations in hardware, software, and implementation, which can compromise reproducibility and deployment stability.^47^ Their limited interpretability further reduces alignment with clinical requirements for transparency and accountability. As a result, conventional models remain more widely used in practice, as they are more stable, more comprehensible, and better suited to settings that require oversight and explainability.^48^

Furthermore, there is no consensus among researchers, including ourselves, regarding model selection and the detailed technical methods. Identifying the optimal ML models and establishing baseline thresholds for risk scores necessitates collaboration between researchers and physicians within a regulated environment, guided by clearly defined standards or recommendations from authoritative bodies. In the UK, progress is being made in the AI healthcare sector, such as the TRIPOD/PROBAST work group.^49^

Another significant limitation concerns model fairness. The CPRD is a primary dataset based in the UK, predominantly including non-Hispanic white individuals.^50^ Although we tested the model on minor ethnicities, issues of fairness persist. It is essential to consider additional socio-economic position (SEP) variables during analysis to enhance the model's fairness.^32^

External validation also poses a challenge. Conducting external validation using datasets from the same country is difficult because the CPRD largely represents the UK general population. Acquiring data from Europe or North America may not adequately account for minor ethnic groups. Datasets from Asia or Africa may be non-existent or of relatively poor quality and size, requiring significant adjustments to fit the original model. This limits the potential for genuine external validation. Open access platforms such as Kaggle^51^ and UCI data repositories^52^ could be beneficial, but currently, no datasets match the required structure and lack of features and CVD outcomes definition. This underscores the necessity for open-source datasets to allow researchers to externally test their models and ensure validity.^13^

Access to advanced health technology remains a significant issue in less developed regions.^53^ While EHR data sharing could benefit these areas, practical challenges in clinical settings persist. Advances in technology and reduced computational costs could facilitate the use of trained models at lower costs. However, it remains uncertain whether this technology will exacerbate health inequalities. Furthermore, there is a pressing need for an open platform that allows researchers to disseminate their findings and validate the work of others, ensuring transparency and replicability. Such a platform would facilitate the development of a consensus on the applicability of data-driven ML-based models in clinical practice.

## Conclusion

This study presents a large-scale analysis of a comprehensive longitudinal EHR dataset, evaluating 10-year health risk prediction for approximately 500,000 CVD patients. We benchmark multiple ML-based survival models against traditional CoxPH models and the widely used QRISK3 score. Our findings demonstrate that ML-based survival models consistently outperform CoxPH, with ensemble-based methods achieving the highest AUC scores of 0.778 for females and 0.738 for males, surpassing NN-based models in both predictive performance and calibration. Given their strong discriminative power, stability, and partial interpretability, ensemble-based approaches emerge as preferable alternatives to NN models for clinical applications. Furthermore, we show that integrating pre-stratified individual risk into ML architectures enhances predictive accuracy, highlighting the value of refining rather than replacing established tools like QRISK3.

## Supplemental Material

sj-docx-1-dhj-10.1177_20552076251408534 - Supplemental material for Benchmarking survival machine learning models for 10-year cardiovascular disease risk prediction using large-scale electronic health records Supplemental material, sj-docx-1-dhj-10.1177_20552076251408534 for Benchmarking survival machine learning models for 10-year cardiovascular disease risk prediction using large-scale electronic health records by Tianyi Liu, Andrew Krentz, Lei Lu, Yanzhong Wang and Vasa Curcin in DIGITAL HEALTH

sj-docx-2-dhj-10.1177_20552076251408534 - Supplemental material for Benchmarking survival machine learning models for 10-year cardiovascular disease risk prediction using large-scale electronic health records Supplemental material, sj-docx-2-dhj-10.1177_20552076251408534 for Benchmarking survival machine learning models for 10-year cardiovascular disease risk prediction using large-scale electronic health records by Tianyi Liu, Andrew Krentz, Lei Lu, Yanzhong Wang and Vasa Curcin in DIGITAL HEALTH

sj-pdf-3-dhj-10.1177_20552076251408534 - Supplemental material for Benchmarking survival machine learning models for 10-year cardiovascular disease risk prediction using large-scale electronic health records Supplemental material, sj-pdf-3-dhj-10.1177_20552076251408534 for Benchmarking survival machine learning models for 10-year cardiovascular disease risk prediction using large-scale electronic health records by Tianyi Liu, Andrew Krentz, Lei Lu, Yanzhong Wang and Vasa Curcin in DIGITAL HEALTH

sj-zip-4-dhj-10.1177_20552076251408534 - Supplemental material for Benchmarking survival machine learning models for 10-year cardiovascular disease risk prediction using large-scale electronic health records Supplemental material, sj-zip-4-dhj-10.1177_20552076251408534 for Benchmarking survival machine learning models for 10-year cardiovascular disease risk prediction using large-scale electronic health records by Tianyi Liu, Andrew Krentz, Lei Lu, Yanzhong Wang and Vasa Curcin in DIGITAL HEALTH
