# Supplementary material for: Benchmarking survival machine learning models for 10-year cardiovascular disease risk prediction using large-scale electronic health records
Source: Digit Health. 2026 Jan 22;12:20552076251408534. doi: 10.1177/20552076251408534 (PMC12833136; doi:10.1177/20552076251408534)
Supplement: sj-docx-1-dhj-10.1177_20552076251408534 - Supplemental material for Benchmarking survival machine learning models for 10-year cardiovascular disease risk prediction using large-scale electronic health records [file sj-docx-1-dhj-10.1177_20552076251408534.docx]

**List of Supplementary Tables**

[Supplementary Table 1 Comorbidities used in the Models 2](#_Toc203164488)

[Supplementary Table 2 CVD Outcomes used in the Models 3](#_Toc203164489)

[Supplementary Table 3 Hyperparameter used in the Models 6](#_Toc203164490)

[Supplementary Table 4 Performance metrics of machine learning models by gender and variables set 7](#_Toc203164491)

| **ICD10** | **Description** | **Disease** | **Category** |
| --- | --- | --- | --- |
| **Atrial fibrillation** | | | |
| I48 | Atrial fibrillation and flutter | Atrial fibrillation | Diagnosis |
| **Erectile dysfunction** | | | |
| N48.4 | Impotence of organic origin | Erectile dysfunction | Diagnosis |
| F52.2 | Failure of genital response | Erectile dysfunction | Possible diagnosis |
| **HIV/AIDS** | | | |
| B20 | Human immunodeficiency virus [HIV] disease resulting in infectious and parasitic diseases | HIV | Diagnosis |
| B21 | Human immunodeficiency virus [HIV] disease resulting in malignant neoplasms | HIV | Diagnosis |
| B22 | Human immunodeficiency virus [HIV] disease resulting in other specified diseases | HIV | Diagnosis |
| B23 | Human immunodeficiency virus [HIV] disease resulting in other conditions | HIV | Diagnosis |
| B24 | Unspecified human immunodeficiency virus [HIV] disease | HIV | Diagnosis |
| F02.4 | Dementia in human immunodeficiency virus [HIV] disease | HIV | Diagnosis |
| R75 | Laboratory evidence of human immunodeficiency virus [HIV] | HIV | Diagnosis |
| Z21 | Asymptomatic human immunodeficiency virus [HIV] infection status | HIV | Diagnosis |
| **Migraine** | | | |
| G43 | Migraine | Migraine | Diagnosis |
| **Rheumatoid arthritis** | | | |
| J99.0 | Rheumatoid lung disease | Rheumatoid arthritis | Diagnosis |
| M05 | Seropositive rheumatoid arthritis | Rheumatoid arthritis | Diagnosis |
| M06 | Other rheumatoid arthritis | Rheumatoid arthritis | Diagnosis |
| **Lupus erythematosus** | | | |
| M32 | Systemic lupus erythematosus | Lupus erythematosus | Diagnosis |
| L93 | Lupus erythematosus | Lupus erythematosus | Diagnosis |

Supplementary Table 1 Comorbidities used in the Models

Supplementary Table 2 CVD Outcomes used in the Models

| **ICD10** | **Description** | **Disease** | **File** |
| --- | --- | --- | --- |
| **Coronary heart disease (angina and myocardial infarction)** | | | |
| I20 | Angina pectoris | Coronary heart disease | CHD |
| I200 | Unstable angina | Unstable angina | Angina unstable |
| I201 | Angina pectoris with documented spasm | Stable angina | Angina stable |
| I208 | Other forms of angina pectoris | Stable angina | Angina stable |
| I209 | Angina pectoris, unspecified | Stable angina | Angina stable |
| I21 | Acute myocardial infarction | Coronary heart disease  Myocardial infarction | CHD/MI |
| I22 | Subsequent myocardial infarction | Coronary heart disease  Myocardial infarction | CHD/MI |
| I23 | Certain current complications following acute myocardial infarction | Coronary heart disease  Myocardial infarction | CHD/MI |
| I24 | Other acute ischaemic heart diseases | Coronary heart disease | CHD |
| I241 | Dressler’s syndrome | Myocardial infarction | MI |
| I25 | Chronic ischaemic heart disease | Coronary heart disease | CHD |
| I250 | Atherosclerotic cardiovascular disease, so described | Coronary heart disease | CHD NOS Death |
| I251 | Atherosclerotic heart disease | Coronary heart disease | CHD NOS Death |
| I252 | Old myocardial infarction | Myocardial infarction | MI |
| I253 | Aneurysm of heart | Coronary heart disease | CHD NOS Death |
| I254 | Coronary artery aneurysm | Coronary heart disease | CHD NOS Death |
| I255 | Ischaemic cardiomyopathy | Coronary heart disease | CHD NOS Death |
| I256 | Silent myocardial ischaemia | Coronary heart disease | CHD NOS Death |
| I258 | Other forms of chronic ischaemic heart disease | Coronary heart disease | CHD NOS Death |
| I259 | Chronic ischaemic heart disease, unspecified | Coronary heart disease | CHD NOS Death |
| **Stroke and TIA** | | | |
| G45.0 | Vertebro-basilar artery syndrome | Transient ischaemic attack | TIA |
| G45.1 | Carotid artery syndrome (hemispheric) | Transient ischaemic attack | TIA |
| G45.2 | Multiple and bilateral precerebral artery syndromes | Transient ischaemic attack | TIA |
| G45.3 | Amaurosis fugax | Transient ischaemic attack | TIA |
| G45.4 | Transient global amnesia | Transient ischaemic attack | TIA |
| G45.8 | Other transient cerebral ischaemic attacks and related syndromes | Transient ischaemic attack | TIA |
| G45.9 | Transient cerebral ischaemic attack, unspecified | Transient ischaemic attack | TIA |
| G46.0 | Middle cerebral artery syndrome | Transient ischaemic attack | TIA |
| G46.1 | Anterior cerebral artery syndrome | Transient ischaemic attack | TIA |
| G46.2 | Posterior cerebral artery syndrome | Transient ischaemic attack | TIA |
| G46.3 | Brain stem stroke syndrome | Stroke | Stroke NOS |
| G46.4 | Cerebellar stroke syndrome | Stroke | Stroke NOS |
| G46.5 | Pure motor lacunar syndrome | Stroke | Stroke NOS |
| G46.6 | Pure sensory lacunar syndrome | Stroke | Stroke NOS |
| G46.7 | Other lacunar syndromes | Stroke | Stroke NOS |
| G46.8 | Other vascular syndromes of brain in cerebrovascular diseases | Stroke | Stroke NOS |
| I63.0 | Cerebral infarction due to thrombosis of precerebral arteries | Stroke | Stroke ischaemic |
| I63.1 | Cerebral infarction due to embolism of precerebral arteries | Stroke | Stroke ischaemic |
| I63.2 | Cerebral infarction due to unspecified occlusion or stenosis of precerebral arteries | Stroke | Stroke ischaemic |
| I63.3 | Cerebral infarction due to thrombosis of cerebral arteries | Stroke | Stroke ischaemic |
| I63.4 | Cerebral infarction due to embolism of cerebral arteries | Stroke | Stroke ischaemic |
| I63.5 | Cerebral infarction due to unspecified occlusion or stenosis of cerebral arteries | Stroke | Stroke ischaemic |
| I63.8 | Other cerebral infarction | Stroke | Stroke ischaemic |
| I63.9 | Cerebral infarction, unspecified | Stroke | Stroke ischaemic |
| I64 | Stroke, not specified as haemorrhage or infarction | Stroke | Stroke NOS |
| I65 | Occlusion and stenosis of precerebral arteries, not resulting in cerebral infarction | Transient ischaemic attack | TIA |
| I66 | Occlusion and stenosis of cerebral arteries, not resulting in cerebral infarction | Transient ischaemic attack | TIA |
| I69.3 | Sequelae of cerebral infarction | Stroke | Stroke ischaemic |
| I69.4 | Sequelae of stroke, not specified as haemorrhage or infarction | Stroke | Stroke NOS |
| **Other (hypertension, heart failure, AAA, and PAD)** | | | |
| I10 | Essential (primary) hypertension | Hypertension | Hypertension |
| I11 | Hypertensive heart disease | Hypertension | Hypertension |
| I11.0 | Hypertensive heart disease with (congestive) heart failure | Heart failure | HF |
| I12 | Hypertensive renal disease | Hypertension | Hypertension |
| I13 | Hypertensive heart and renal disease | Hypertension | Hypertension |
| I13.0 | Hypertensive heart and renal disease with (congestive) heart failure | Heart failure | HF |
| I13.2 | Hypertensive heart and renal disease with both (congestive) heart failure and renal failure | Heart failure | HF |
| I15 | Secondary hypertension | Hypertension | Hypertension |
| I50 | Heart failure | Heart failure | HF |
| I713 | Abdominal aortic aneurysm, ruptured | Abdominal aortic aneurysm | AAA |
| I714 | Abdominal aortic aneurysm, without mention of rupture | Abdominal aortic aneurysm | AAA |
| I715 | Thoracoabdominal aortic aneurysm, ruptured | Abdominal aortic aneurysm | AAA |
| I716 | Thoracoabdominal aortic aneurysm, without mention of rupture | Abdominal aortic aneurysm | AAA |
| I718 | Aortic aneurysm of unspecified site, ruptured | Abdominal aortic aneurysm | AAA |
| I719 | Aortic aneurysm of unspecified site, without mention of rupture | Abdominal aortic aneurysm | AAA |
| I731 | Thromboangiitis obliterans [Buerger] | Peripheral arterial disease | PAD |
| I738 | Other specified peripheral vascular diseases | Peripheral arterial disease | PAD |
| I739 | Peripheral vascular disease, unspecified | Peripheral arterial disease | PAD |
| I743 | Embolism and thrombosis of arteries of lower extremities | Peripheral arterial disease | PAD |
| I744 | Embolism and thrombosis of arteries of extremities, unspecified | Peripheral arterial disease | PAD |
| I745 | Embolism and thrombosis of iliac artery | Peripheral arterial disease | PAD |

Supplementary Table 3 Hyperparameter used in the Models

| **Models (package)** | **Gender** | **Hyperparameters** |
| --- | --- | --- |
| CoxPH (lifelines) | Male | {'penalizer': 0.016492995445404557, 'l1_ratio': 0.044399866137556865} |
|  | Female | {'penalizer': 0.03658779898446701, 'l1_ratio': 0.007825614656993721} |
| CoxPH (sksurv) | Male | {'alpha': 9.008292046338946, 'tol': 1.4285676124002436e-06} |
|  | Female | {'alpha': 0.01444525102276306, 'tol': 1.461896279370496e-05} |
| RSF (sksurv) | Male | {'n_estimators': 200, 'max_depth': 20, 'min_samples_split': 2, 'min_samples_leaf': 1} |
|  | Female | {'n_estimators': 200, 'max_depth': 20, 'min_samples_split': 2, 'min_samples_leaf': 1} |
| GBSA (sksurv) | Male | {'learning_rate': 0.13353819088790583, 'n_estimators': 200, 'max_depth': 5, 'min_samples_split': 10, 'min_samples_leaf': 5} |
|  | Female | {'learning_rate': 0.07475992999956503, 'n_estimators': 200, 'max_depth': 7, 'min_samples_split': 10, 'min_samples_leaf': 5} |
| XGBS (xgboost) | Male | {'learning_rate': 0.032017635817134925, 'aft_loss_distribution': 'logistic', 'aft_loss_distribution_scale': 1.4864726581962449, 'max_depth': 6, 'lambda': 0.00011255221108970817, 'alpha': 0.00013359696956465752, 'objective': 'survival:aft', 'eval_metric': 'aft-nloglik', 'tree_method': 'hist'} |
|  | Female | {'learning_rate': 0.015246158270734918, 'aft_loss_distribution': 'logistic', 'aft_loss_distribution_scale': 1.2848800241869007, 'max_depth': 7, 'lambda': 1.5352295063469784e-06, 'alpha': 0.0014632389176876897, 'objective': 'survival:aft', 'eval_metric': 'aft-nloglik', 'tree_method': 'hist'} |
| DeepSurv (pycox) | Male | {'num_nodes': '128-64', 'dropout': 0.2550230576447221, 'batch_norm': False, 'lr': 0.0009920562866268196} |
|  | Female | {'num_nodes': '128-64', 'dropout': 0.46974947078209456, 'batch_norm': True, 'lr': 0.0069782812651260325} |
| DeepHit (pycox) | Male | {'num_nodes': '32-32', 'dropout': 0.24064424648301141, 'batch_norm': True, 'lr': 0.0007707153486748526, 'alpha': 0.6286845802338294} |
|  | Female | {'num_nodes': '32-32', 'dropout': 0.25070737552220296, 'batch_norm': True, 'lr': 0.0007957559117863843, 'alpha': 0.619619038800538} |

Supplementary Table 4 Performance metrics of machine learning models by gender and variables set

|  | | Male | | | | | | | Female | | | | | | |
| --- | --- | --- | --- | --- | --- | --- | --- | --- | --- | --- | --- | --- | --- | --- | --- |
|  |  | Baseline threshold (%) | Accuracy | Specificity | Recall | F1 | AUROC (95% CI) | Brier score | Baseline threshold (%) | Accuracy | Specificity | Recall | F1 | AUROC (95% CI) | Brier score |
| CoxPH (lifelines) | QRISK3 set | 9.03  9.02  9.35  8.44 | 0.654  0.658  0.670  0.656 | 0.652  0.656  0.672  0.654 | 0.672  0.682  0.657  0.677 | 0.297  0.303  0.302  0.262 | 0.721 (0.718, 0.724)  0.724 (0.718, 0.730)  0.721 (0.715, 0.727)  0.723 (0.716, 0.729) | 0.091  0.092  0.091  0.078 | 5.39  5.61  5.74  4.85 | 0.660  0.677  0.689  0.679 | 0.656  0.676  0.688  0.677 | 0.719  0.697  0.708  0.725 | 0.218  0.222  0.231  0.183 | 0.748 (0.745, 0.752)  0.749 (0.741, 0.757)  0.758 (0.750, 0.765)  0.766 (0.760, 0.774) | 0.058  0.058  0.058  0.045 |
|  | Expanded stratified risk set | 10.06  9.97  10.15  9.77 | 0.656  0.655  0.661  0.658 | 0.653  0.651  0.660  0.656 | 0.679  0.694  0.674  0.677 | 0.300  0.303  0.302  0.264 | 0.723 (0.720, 0.726)  0.726 (0.720, 0.733)  0.724 (0.717, 0.730)  0.725 (0.719, 0.731) | 0.091  0.091  0.091  0.078 | 6.10  6.09  5.95  5.68 | 0.680  0.681  0.675  0.692 | 0.679  0.679  0.671  0.691 | 0.700  0.705  0.731  0.717 | 0.224  0.226  0.229  0.188 | 0.750 (0.747, 0.754)  0.752 (0.744, 0.760)  0.760 (0.752, 0.767)  0.769 (0.762, 0.776) | 0.058  0.058  0.058  0.045 |
| CoxPH (sksurv) | QRISK3 set | 9.00  9.06  9.02  8.34 | 0.655  0.662  0.657  0.659 | 0.653  0.660  0.655  0.657 | 0.671  0.676  0.674  0.675 | 0.297  0.303  0.300  0.263 | 0.721 (0.718, 0.724)  0.724 (0.717, 0.730)  0.721 (0.715, 0.727)  0.722 (0.716, 0.729) | 0.091  0.092  0.091  0.078 | 5.40  5.26  5.76  4.60 | 0.673  0.663  0.576  0.677 | 0.671  0.659  0.699  0.675 | 0.701  0.715  0.694  0.725 | 0.220  0.219  0.233  0.182 | 0.748 (0.744, 0.751)  0.748 (0.741, 0.756)  0.757 (0.750, 0.765)  0.766 (0.759, 0.774) | 0.058  0.058  0.058  0.045 |
|  | Expanded stratified risk set | 9.89  10.52  10.71  9.77 | 0.641  0.672  0.676  0.662 | 0.635  0.672  0.679  0.661 | 0.697  0.666  0.654  0.671 | 0.297  0.306  0.305  0.264 | 0.723 (0.719, 0.725)  0.725 (0.719, 0.731)  0.723 (0.717, 0.730)  0.724 (0.717, 0.730) | 0.091  0.091  0.091  0.078 | 6.10  6.31  6.18  5.36 | 0.675  0.687  0.682  0.688 | 0.673  0.687  0.680  0.686 | 0.705  0.693  0.717  0.717 | 0.223  0.226  0.229  0.186 | 0.750 (0.746, 0.753)  0.751 (0.743, 0.759)  0.759 (0.751, 0.766)  0.768 (0.761, 0.776) | 0.058  0.058  0.058  0.045 |
| RSF | QRISK3 set | 14.03  10.04  9.32  11.79 | 0.831  0.669  0.634  0.639 | 0.842  0.667  0.621  0.637 | 0.745  0.679  0.735  0.667 | 0.496  0.313  0.308  0.250 | 0.889 (0.884, 0.894)  0.737 (0.723, 0.752)  0.732 (0.717, 0.746)  0.704 (0.698, 0.711) | 0.069  0.090  0.090  0.080 | 13.23  5.86  7.36  6.81 | 0.964  0.675  0.743  0.660 | 0.968  0.752  0.683  0.659 | 0.915  0.752  0.683  0.683 | 0.772  0.235  0.260  0.167 | 0.965 (0.962, 0.968)  0.763 (0.746, 0.779)  0.771 (0.755, 0.786)  0.730 (0.723, 0.738) | 0.038  0.056  0.056  0.046 |
|  | Expanded stratified risk set | 15.94  10.45  10.53  12.28 | 0.941  0.662  0.663  0.638 | 0.950  0.658  0.659  0.645 | 0.867  0.694  0.699  0.684 | 0.764  0.313  0.316  0.254 | 0.934 (0.930, 0.937)  0.739 (0.724, 0.753)  0.738 (0.723, 0.752)  0.707 (0.701, 0.713) | 0.057  0.089  0.088  0.080 | 13.8  6.59  6.85  7.72 | 0.979  0.709  0.719  0.700 | 0.985  0.708  0.720  0.675 | 0.889  0.721  0.713  0.669 | 0.850  0.247  0.252  0.181 | 0.959 (0.955, 0.962)  0.776 (0.758, 0.792)  0.778 (0.762, 0.793)  0.744 (0.737, 0.752) | 0.031  0.055  0.055  0.046 |
| GBSA | QRISK3 set | 10.22  10.02  9.59  10.81 | 0.685  0.660  0.637  0.651 | 0.681  0.659  0.629  0.651 | 0.716  0.667  0.696  0.649 | 0.336  0.304  0.299  0.251 | 0.777 (0.770, 0.784)  0.722 (0.708, 0.737)  0.719 (0.705, 0.734)  0.705 (0.698, 0.711) | 0.084  0.093  0.092  0.082 | 5.52  5.47  5.30  5.34 | 0.702  0.684  0.674  0.668 | 0.699  0.685  0.671  0.666 | 0.745  0.676  0.710  0.704 | 0.249  0.221  0.224  0.174 | 0.806 (0.798, 0.813)  0.737 (0.718, 0.755)  0.749 (0.733, 0.766)  0.740 (0.733, 0.747) | 0.052  0.058  0.058  0.047 |
|  | Expanded stratified risk set | 10.67  9.97  10.06  11.00 | 0.675  0.658  0.654  0.659 | 0.673  0.655  0.650  0.638 | 0.686  0.680  0.689  0.677 | 0.319  0.306  0.307  0.264 | 0.748 (0.742, 0.755)  0.722 (0.708, 0.736)  0.728 (0.713, 0.742)  0.725 (0.719, 0.731) | 0.090  0.092  0.091  0.078 | 5.82  5.65  5.36  5.40 | 0.719  0.694  0.668  0.682 | 0.716  0.694  0.664  0.673 | 0.762  0.700  0.717  0.722 | 0.265  0.233  0.223  0.184 | 0.823 (0.816, 0.829)  0.752 (0.734, 0.769)  0.751 (0.735, 0.766)  0.761 (0.753, 0.768) | 0.054  0.058  0.058  0.046 |
| XGBS | QRISK3 set | 6.67  5.27  5.10  4.71 | 0.737  0.662  0.652  0.654 | 0.739  0.659  0.647  0.654 | 0.725  0.686  0.697  0.654 | 0.375  0.306  0.304  0.255 | 0.817 (0.814, 0.819)  0.730 (0.723, 0.737)  0.732 (0.726, 0.739)  0.708 (0.702, 0.715) | 0.084  0.093  0.092  0.080 | 2.92  4.82  2.00  1.14 | 0.796  0.689  0.708  0.664 | 0.798  0.689  0.710  0.662 | 0.763  0.694  0.683  0.705 | 0.330  0.227  0.236  0.172 | 0.872 (0.870, 0.875)  0.757 (0.748, 0.764)  0.765 (0.758, 0.772)  0.739 (0.732, 0.747) | 0.051  0.059  0.059  0.047 |
|  | Expanded stratified risk set | 4.09  3.39  3.27  3.78 | 0.724  0.669  0.659  0.652 | 0.722  0.667  0.655  0.650 | 0.741  0.683  0.692  0.670 | 0.369  0.310  0.306  0.258 | 0.814 (0.812, 0.817)  0.733 (0.727, 0.740)  0.736 (0.730, 0.743)  0.715 (0.709, 0.721) | 0.088  0.095  0.095  0.081 | 3.22  2.70  2.58  2.71 | 0.730  0.682  0.677  0.670 | 0.729  0.682  0.674  0.667 | 0.747  0.691  0.722  0.720 | 0.267  0.223  0.228  0.178 | 0.824 (0.821, 0.827)  0.753 (0.745, 0.761)  0.762 (0.754, 0.769)  0.754 (0.746, 0.761) | 0.055  0.059  0.059  0.046 |
| DeepSurv | QRISK3 set | 10.85  10.77  10.69  9.47 | 0.669  0.664  0.658  0.641 | 0.666  0.663  0.655  0.636 | 0.691  0.675  0.683  0.687 | 0.312  0.304  0.303  0.257 | 0.746 (0.743, 0.749)  0.728 (0.721, 0.734)  0.727 (0.721, 0.734)  0.716 (0.709, 0.722) | 0.088  0.090  0.090  0.078 | 6.13  5.97  6.13  5.26 | 0.684  0.676  0.681  0.688 | 0.683  0.674  0.680  0.687 | 0.697  0.706  0.698  0.711 | 0.225  0.223  0.224  0.185 | 0.752 (0.748, 0.756)  0.752 (0.744, 0.759)  0.751 (0.743, 0.759)  0.764 (0.756, 0.771) | 0.058  0.058  0.058  0.045 |
|  | Expanded stratified risk set | 10.75  10.26  10.15  9.92 | 0.673  0.655  0.649  0.652 | 0.671  0.650  0.643  0.649 | 0.688  0.688  0.695  0.679 | 0.314  0.302  0.301  0.260 | 0.747 (0.744, 0.750)  0.730 (0.724, 0.736)  0.727 (0.721, 0.733)  0.722 (0.715, 0.728) | 0.088  0.090  0.090  0.079 | 6.81  6.80  6.98  6.31 | 0.672  0.671  0.688  0.688 | 0.670  0.669  0.686  0.687 | 0.705  0.706  0.706  0.718 | 0.221  0.221  0.230  0.186 | 0.749 (0.745, 0.752)  0.748 (0.740, 0.756)  0.758 (0.750, 0.765)  0.765 (0.758, 0.772) | 0.058  0.058  0.058  0.045 |
| DeepHit | QRISK3 set | 8.96  9.23  9.96  7.80 | 0.664  0.641  0.646  0.660 | 0.635  0.647  0.651  0.659 | 0.677  0.691  0.704  0.669 | 0.305  0.295  0.302  0.263 | 0.730 (0.727, 0.734)  0.720 (0.713, 0.727)  0.729 (0.722, 0.735)  0.722 (0.715, 0.728) | 0.097  0.098  0.097  0.079 | 4.99  4.73  4.69  4.07 | 0.675  0.682  0.707  0.694 | 0.683  0.698  0.687  0.693 | 0.717  0.703  0.691  0.706 | 0.225  0.226  0.238  0.186 | 0.759 (0.756, 0.763)  0.754 (0.747, 0.762)  0.762 (0.755, 0.769)  0.766 (0.759, 0.773) | 0.058  0.058  0.058  0.045 |
|  | Expanded stratified risk set | 8.57  8.11  8.56  7.34 | 0.657  0.660  0.658  0.653 | 0.662  0.636  0.628  0.650 | 0.679  0.685  0.679  0.683 | 0.301  0.305  0.301  0.263 | 0.726 (0.723, 0.729)  0.728 (0.721, 0.734)  0.725 (0.719, 0.732)  0.726 (0.719, 0.732) | 0.098  0.098  0.098  0.079 | 5.14  5.13  5.14  5.10 | 0.685  0.669  0.685  0.682 | 0.678  0.665  0.674  0.679 | 0.710  0.722  0.718  0.728 | 0.229  0.224  0.231  0.185 | 0.762 (0.759, 0.766)  0.756 (0.748, 0.764)  0.763 (0.756, 0.771)  0.768 (0.761, 0.775) | 0.058  0.058  0.058  0.045 |

**NB:** In each cell, Train: first value; Validation: second value; Test: third value; ‘Spatial validation’: fourth value
