# Supplementary material for: Benchmarking survival machine learning models for 10-year cardiovascular disease risk prediction using large-scale electronic health records
Source: Digit Health. 2026 Jan 22;12:20552076251408534. doi: 10.1177/20552076251408534 (PMC12833136; doi:10.1177/20552076251408534)
Supplement: sj-docx-2-dhj-10.1177_20552076251408534 - Supplemental material for Benchmarking survival machine learning models for 10-year cardiovascular disease risk prediction using large-scale electronic health records [file sj-docx-2-dhj-10.1177_20552076251408534.docx]

**Supplementary Material: Sample Size Justification for Model Development**

To ensure methodological rigour and reduce the risk of overfitting in developing our time-to-event machine learning models for 10-year CVD risk prediction, we conducted a minimum sample size calculation based on the framework proposed by Riley et al. (2019), which provides validated criteria for sample size estimation in the development of prognostic models with time-to-event outcomes. This framework accounts for the number of predictor parameters (degrees of freedom), anticipated outcome incidence, follow-up duration, a pre-specified shrinkage factor, and the proportion of variance explained by the model.

For our primary Cox model, we assumed 35 predictor parameters based on the final set of features used in model development. We selected a conservative shrinkage factor of 0.9 to constrain potential overfitting and ensure shrinkage-adjusted predictor effects. The expected 10-year cumulative incidence of CVD was conservatively set at 5%, which is slightly lower than the observed incidence of 7.5% in our training dataset. Mean follow-up duration was assumed to be 10 years. The proportion of variance explained by the model (R²ₚₑₐₖ) was set to 0.15, a commonly used conservative default in prognostic modelling when a priori estimates are unavailable.

Using the closed-form sample size formula described in Riley et al. (2019, Part II), the minimum required sample size was calculated to be 16,242 individuals, corresponding to a minimum of 812 CVD events and an events-per-parameter ratio of approximately 23.2. These thresholds provide adequate statistical power and support for stable model calibration and generalisability.

Our training dataset comprised 512,560 patient records, among which 38,442 experienced a CVD event within the 10-year prediction window. This yields an events-per-parameter ratio of approximately 1,098, substantially exceeding the minimum required thresholds by a wide margin. This ensures precise effect estimation, reliable calibration, and low risk of model overfitting. Notably, while our observed event incidence (7.5%) is higher than the conservative 5% used in the sample size calculation, we retained the lower estimate to ensure the robustness of our justification.

Finally, our empirical incidence is consistent with real-world UK population estimates, where the British Heart Foundation reports that approximately 11% of adults are living with CVD and the annual incidence for major events such as MI or stroke is approximately 1.5 per 1,000. This supports the external plausibility of our modelling assumptions.

**References**

Riley RD, Snell KI, Ensor J, et al. Minimum sample size for developing a multivariable prediction model: Part II – binary and time-to-event outcomes. Stat Med. 2019;38(7):1276–1296.

British Heart Foundation. UK Factsheet, 2023. <https://www.bhf.org.uk/what-we-do/our-research/heart-statistics>
